# Understanding COVID-19 Vaccine Hesitancy in the United States: A Systematic Review

**DOI:** 10.3390/vaccines12070747

**Published:** 2024-07-06

**Authors:** Godspower Nwachukwu, Alaa Rihan, Esther Nwachukwu, Ndukwe Uduma, Kimberly S. Elliott, Yordanos M. Tiruneh

**Affiliations:** 1 Department of Public Health, School of Health Professions, University of Texas at Tyler Health Science Center, Tyler, TX 75708, USA; gnwachukwu@patriots.uttyler.edu; 2 Department of Preventive Medicine and Population Health, School of Medicine, University of Texas at Tyler Health Science Center, Tyler, TX 75708, USA; yordanos.tiruneh@uttyler.edu; 3 Department of Biology, University of Texas at Tyler, Tyler, TX 75799, USA; enwachukwu@patriots.uttyler.edu (E.N.); nuduma@patriots.uttyler.edu (N.U.); 4 Department of Health Policy, Economics and Management, School of Health Professions, University of Texas at Tyler Health Science Center, Tyler, TX 75708, USA; kimberly.elliott@uthct.edu; 5 Department of Internal Medicine, Division of Infectious Diseases, University of Texas Southwestern Medical Center, Dallas, TX 75390, USA

**Keywords:** COVID-19 pandemic, vaccination, vaccine hesitancy, vaccine acceptance, vaccine perceptions, vaccine uptake, mistrust, misinformation, vaccine safety, United States

## Abstract

The COVID-19 pandemic has presented the importance of vaccination as a pivotal strategy for controlling its spread. However, vaccine hesitancy poses a significant barrier to achieving widespread immunization in the United States. This systematic review utilizes the 5C model to examine the factors contributing to hesitancy, which include confidence in vaccines, complacency about disease risk, calculations of individual benefit, convenience of vaccination, and collective responsibility for the protection of others. Methods: We conducted a comprehensive search across several relevant databases and the gray literature, identifying 544 studies that used quantitative and qualitative methods to explore COVID-19 vaccine hesitancy in the general U.S. population. Results: This review identifies a complex interplay of factors affecting hesitancy, such as concerns over vaccine safety and efficacy, misinformation and conspiracy theories, demographic variables, and socioeconomic conditions. Key strategies for increasing vaccine uptake include transparent and effective communication along with proactive community engagement. Conclusions: To effectively mitigate vaccine hesitancy, it is crucial to understand its multifaceted causes. Tailored interventions that consider socioeconomic and cultural contexts and prioritize clear communication, community involvement, and specific strategies to address unique concerns can enhance vaccine acceptance.

## 1. Introduction

The COVID-19 pandemic has emerged as an unparalleled global health crisis. According to the World Health Organization (WHO) report in September 2023, worldwide COVID-19 cases surpassed 770 million, resulting in a death toll of 6.9 million [1]. The pandemic has deeply affected various aspects of life, prompting the global community to acknowledge the immediate need for effective safety measures, and the COVID-19 vaccine emerged as a central strategy in response. The past three years witnessed an unprecedented global collaboration to accelerate the development and distribution of COVID-19 vaccines. Prominent organizations such as Pfizer, Moderna, and the COVAX Facility of the WHO have been at the forefront of advocating for fair distribution of vaccines [2]. Additionally, the U.S. initiative, Operation Warp Speed, further expedited vaccine availability [3]. These collaborative efforts culminated in the administration of over 13 billion doses worldwide, with the U.S. accounting for over 668 million administered doses [1]. As of May 2023, 81.4% of eligible Americans had received at least one dose of the COVID-19 vaccine, reflecting the multifaceted dynamics of vaccine perceptions [4]. Factors such as fear, mistrust, misinformation, and lack of knowledge have stymied vaccine uptake [5]. The swift development of the COVID-19 vaccine has also raised concerns about its safety, efficacy, and potential long-term effects. While vaccine hesitancy is not a new concept, hesitancy towards the COVID-19 vaccine is a phenomenon that must be examined as this virus will remain a public health issue for the foreseeable future.

Despite aggressive efforts by countries, including the United States, in vaccine research, development, and distribution, vaccine hesitancy has hindered widespread acceptance. The WHO has identified this reluctance or outright refusal to vaccinate, despite available services, as one of the top threats to global health [6].

In the U.S., this challenge has been particularly pronounced, by ideological polarization surrounding health decisions and the spread of misinformation regarding the disease and the vaccine. Factors such as political affiliations, cultural beliefs, and concerns about the rapid development of the vaccine have also played a part in shaping public sentiment [7,8,9,10,11].

**Figure 1:**
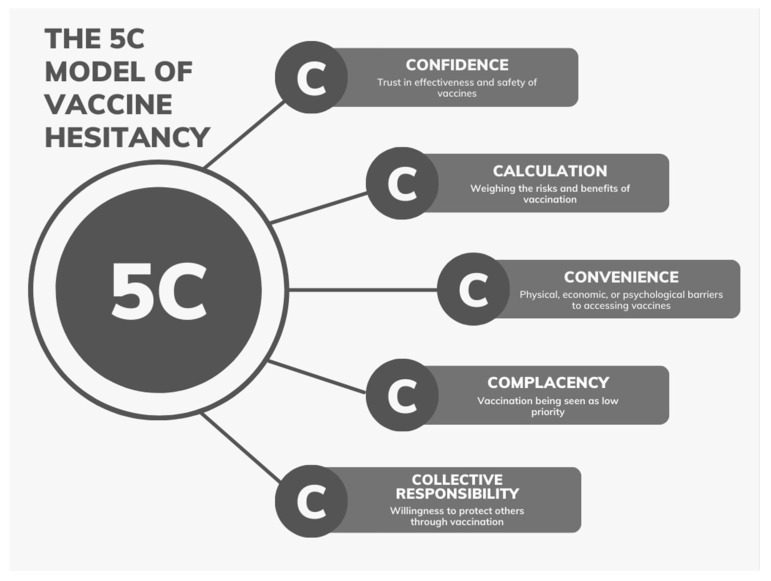
Schematic representation of the 5C model illustrating the interplay between confidence, complacency, convenience, calculation, and collective responsibility in shaping vaccine hesitancy.

The 5C model is among the most common tools used in the field of vaccination behavior, alongside the health belief model (HBM) and the theory of planned behavior (TPB) [12]. The 5C theoretical model breaks down the concept of hesitancy into five distinct categories: (1) complacency—reflecting a perceived low risk of the disease; (2) convenience—pertaining to the accessibility and ease of getting vaccinated; (3) confidence—representing trust in the vaccine’s safety and efficacy; (4) calculation—involving individual evaluation of perceived risks versus benefits; and (5) collective responsibility—emphasizing the broader role of vaccination in safeguarding the community [13]. The insights from the 5C model can guide the development of strategies to bolster vaccine uptake and counter hesitancy. Figure 1 showcases the 5C model, which is based on the concepts mentioned prior. Through this systematic review, we aim to explore the complex dynamics of COVID-19 vaccine hesitancy in the U.S., utilizing the 5C model as our conceptual framework.

## 2. Materials and Methods

### 2.1. Data Sources and Search Strategies

This systematic review followed the preferred reporting items for systematic reviews and meta-analyses (PRISMA) guidelines. We searched 20 different electronic databases, including Ebscohost databases such as APA Psycarticles, APA PsycInfo, CINAHL, Health Source: Nursing/Academic Edition, Medline, and Psychology and Behavioral Sciences Collection. Additionally, ProQuest databases, including Biological Science Collection, Health and Medical Collection, Psychology Database, and Public Health Database, were searched. Elsevier’s Science Direct and Scopus, Cochrane’s COVID-19 Study Register and Cochrane Library, and the gray literature sources like Google Scholar were also consulted. Furthermore, other databases such as JSTOR, PUBMED, Springerlink, Web of Science, and Wiley Online Library were included to ensure a comprehensive and thorough search of the relevant literature. We selected peer-reviewed articles published in English within the United States between 2022 and 2023, specifically targeting vaccine hesitancy and acceptance. This time frame was chosen strategically to complement existing research, as a prior systematic review on COVID-19 vaccine hesitancy already covered data from articles published in 2020 and 2021 [14]. The search strategy incorporated a combination of medical subject headings (MeSH) and keywords such as “COVID-19 vaccine”, “vaccine hesitancy”, “vaccine acceptance”, and “United States’’. Information regarding the materials and the MeSH terms can be found in Appendix A Table A1 and Table A2.

### 2.2. Inclusion and Exclusion Criteria

The search for this study was conducted initially in July 2023, which involved gathering studies from 20 diverse databases. These studies were extracted as RIS files and imported into Zotero, a reference manager application. When the studies were imported, they were sorted based on the type of study they were such as book sections, blog posts, and magazine articles. Table A3 goes into detail on the number and types of materials imported into Zotero. The non-journal articles were removed, leaving only the studies that were journal articles. Although some of the data from the non-journal could provide pertinent information regarding public sentiment, their removal was justified due to the lack of both scientific rigor and peer review that journal articles would undergo, which would ensure the article’s validity, reliability, and credibility. Using journal articles in this study also helped with consistency of the data due to academic journals following standardized formats in presentation of research findings. Sources such as magazine and blog posts could also be subjective and opinionated based on the author’s personal ideals, which creates bias. After removing duplicate articles, the remaining studies were uploaded to Covidence, an online software for systematic reviews, where they were further assessed based on titles and abstracts (app.covidence.org, accessed on 21 August 2023). The list was updated in November 2023, with additional databases searched and the same screening process employed, utilizing Covidence for organization. Eligibility criteria included peer-reviewed articles conducted in the U.S., published between 2022 and 2023, with full text accessible during the review, and presenting data concerning COVID-19 vaccine hesitancy or acceptance. Exclusion criteria comprised editorials, reviews, or opinion pieces, research not executed in the U.S. or not written in English, and studies with unclear methodologies or insufficient data.

### 2.3. Review Process

The review process was structured and conducted to ensure the study analysis was both thorough and unbiased. All members of the study team took part in appraising the articles to assess the relevance of the studies to this study’s overall objectives and extract data. The articles were split among the study team, and all of the articles included in this study were reviewed independently by at least two team members to reduce the possibility of selection bias. This review was conducted on Covidence, an online tool used for collaboration of systematic reviews. Articles collected from the databases were uploaded to Covidence and underwent title and abstract screening, which had each article evaluated individually. If there was any discrepancy regarding the eligibility of an article, the article would be placed in a conflict list where a third reviewer would be able to make a final vote on that article. After title and abstract screening, the articles that met the criteria were moved to a full text review. This review process was conducted the same way as the initial review, with two reviewers screening the full text of an article, and a third reviewer reviewing conflicting or controversial articles. Finally, data extraction was conducted on articles that qualified for inclusion in the full text review.

### 2.4. Data Extraction

Data extraction was conducted using standardized forms designed to capture essential information, including the year of publication, study setting, design, population demographics, vaccine acceptance rates, and key findings related to vaccine hesitancy. The 5C model was a variable used in this study to better evaluate the data presented in this study. The model has been used in a variety of articles to monitor the varying status of vaccination in distinct groups. Terms from the 5C model were applied to each study based on the primary hesitancy factor listed for that article. Other variables included in this study were extracted individually from each study and individually placed into three separate groups based on the data. The first group was study characteristics, which included year of publication, which was grouped based on either being published in 2022 or 2023. Study setting was categorized as web-based, in-person, telephone-based, or a combination of the different types (hybrid). Study design included cross-sectional survey, mixed method design, qualitative studies, longitudinal studies, and others tagged as miscellaneous such as observational, ecological, and descriptive studies that were few in count. Population type was broken down into various groups including U.S. adults, ethnic and racial minority populations, healthcare workers, people with specific medical conditions, U.S. parents, university population, pregnant or postpartum women, and age-specific studies. Sample size ranges split the studies based on the sizes of their samples, ranging from studies having between 0 and 100 participants, to those having over 100,000. The second group focused on the geographical distribution of the studies throughout the United States, which grouped the studies based on the location that the study was conducted in. The third group focused on the vaccine-related characteristics from the study. The 5C model was one of the variables included in this group, which categorized the articles based on applying each of the 5C terms (confidence, convenience, calculation, complacency, and collective responsibility) to the hesitancy factors identified in the articles. Multiple 5C terms could be used to describe a single article. The reported vaccine uptake rate was the second variable that was included in this section. The percent ranges of vaccine acceptance were categorized by the percent ranges with some ranging from 0 to 10% and 11 to 20%, to percentages ranging up to 81–90% or 91–100%. Hesitancy predictors were also collected from the articles, which included a variety of terms such as health concerns, speed of vaccine development/safety concerns, mistrust and compliance, and convenience. Uptake factors was the final variable that was collected from the groups, which had split the data based on terms such as trust and confidence, community and social factors, demographics and identity, healthcare provider recommendations, health concerns, and psychological factors. Quality assessment procedures were implemented to evaluate the credibility of the included studies based on their methodologies and reported findings. Some of the steps used for credibility assessment included having criteria for evaluation from the study requiring clarity and relevance from the findings. Having a reviewer process was also important, with each study being evaluated by multiple reviewers. Critical evaluation involved studies with apparent methodological shortcomings or inconsistencies in their results having to undergo critical evaluation by the multiple reviewers, with only those demonstrating reliable methodologies and consistent results being considered for subsequent analysis. Having inclusion criteria also helped so that only studies that demonstrated reliable methodologies and consistent results would be considered for subsequent analysis, which caused the studies that failed to meet the criteria to be excluded from further consideration. Data synthesis and analysis involved the narrative synthesis of findings from the incorporated studies, with a specific emphasis on discerning the factors influencing COVID-19 vaccine hesitancy and acceptance in the United States. The synthesis process was started by extracting data from each of the articles, followed by organization of the data into the different characteristic groups. The narrative synthesis integrated the overall findings from the various findings to provide a comprehensive overview of the factors that were able to influence both hesitancy and acceptance in the U.S., which included summarizing key points and drawing connections between the different articles. Given that the review relied on previously published data, IRB and ethical approval were deemed unnecessary.

## 3. Results

### 3.1. Study Selection Process

**Figure 2:**
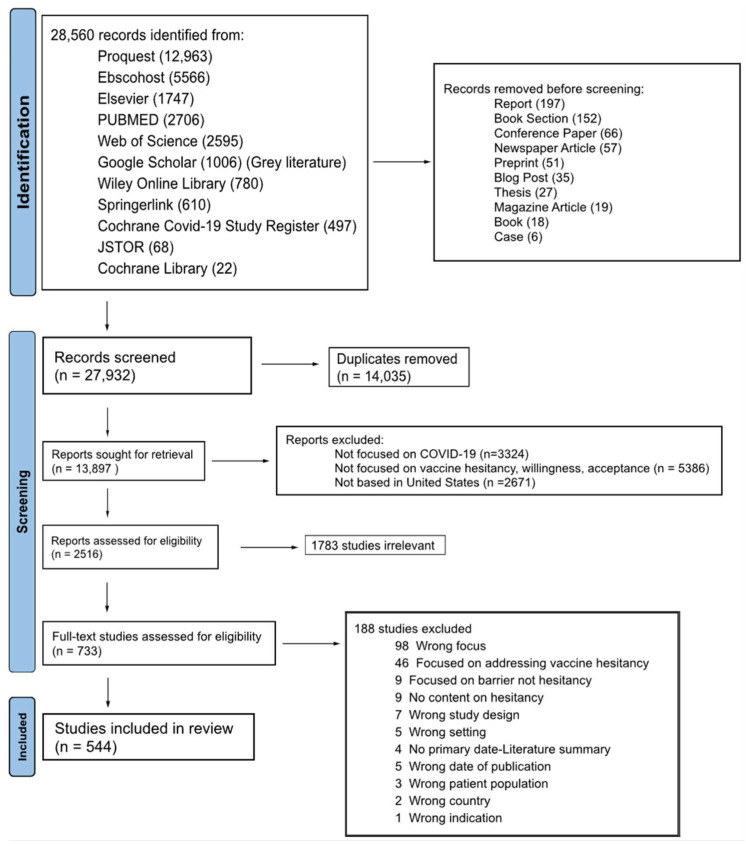
PRISMA (preferred reporting items for systematic reviews and meta-analyses) flow diagram showing the selection process for studies included in the systematic review.

We first identified 28,560 studies through database searching, of which 27,932 were journal articles. After screening based on title and abstract relevancy, 2516 were further assessed for eligibility according to our inclusion criteria, which focused on COVID-19 vaccine hesitancy in the United States. Ultimately, 544 studies met all criteria and were included in the final systematic review. The complete search and selection process for this study is visually summarized in the PRISMA flow diagram [9] (Figure 2).

### 3.2. Study Characteristics

Table 1 describes the study characteristics, including variables such as year of publication, study setting, study design, and population type.

This review analyzed articles published over two consecutive years. Table A3 shows that most of the studies included in this review were published in 544 studies, accounting for 58.82% of the total articles. In 2023, there was a decrease in publications, comprising 41.18% of the studies. Specifically, there were 320 publications in 2022 and 224 in 2023. This represents a 30% decrease in the number of publications from 2022 to 2023.

Many of the studies were web-based or conducted online, and these studies made up 70.59% of the studies analyzed. In-person studies made up 14.15% of the studies and were often conducted in hospital settings, providing valuable insight through direct engagement. Studies that used multiple modalities of data collection, which combined web-based, in-person, or telephone-based approaches, made up 8.27% of the studies included in this review. These studies aimed to gain a comprehensive understanding of participants’ attitudes and behaviors towards vaccination by using diverse data collection methods. Telephone-based surveys, representing 6.99% of the studies, were the least used method.

This systematic review encompassed diverse study designs, predominantly cross-sectional surveys, which constituted 73.35% of the total studies analyzed. Additionally, mixed methods (8.09%) and qualitative methods (6.80%) were included, reflecting the varied methodologies used to explore vaccine hesitancy. Longitudinal and cohort studies were also included in this study (7.72%). Both studies involved long-term data collection, with longitudinal studies involving tracking changes in vaccine attitudes over time and cohort studies following groups over periods of time to observe changes and outcomes that may relate to hesitancy.

**Figure 3:**
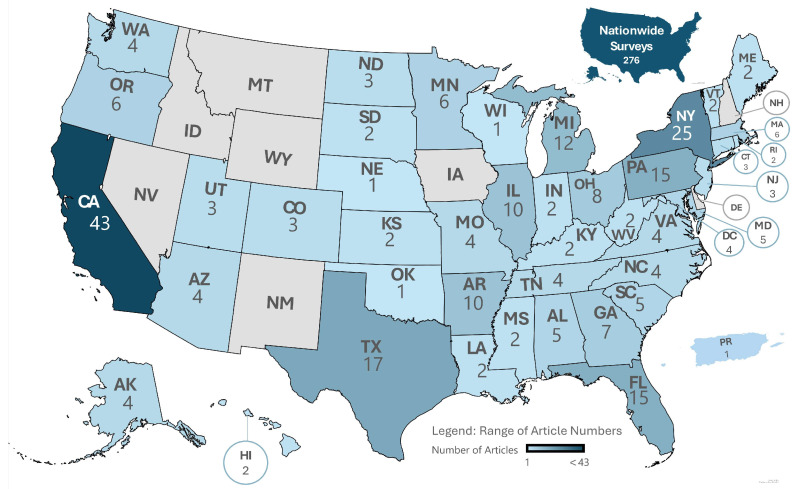
Geographic distribution of vaccine hesitancy studies across the United States.

This review included a variety of populations, enhancing the overall understanding of vaccine hesitancy from different groups. The study population for about a third of the studies was general U.S. adults (32.35%), with some studies focusing on specific state/s and others being all inclusive. Figure 3 depicts the distribution of the studies throughout the country, representing each individual state and territory relevant to this study. Ethnic and racial minority populations represented 14.71% of the total studies, highlighting the importance of examining vaccine perceptions among diverse racial and ethnic groups. Healthcare workers were the focus of 11.95% of the studies reviewed, underscoring their critical role in the healthcare system and their influence on public vaccine acceptance. People with specific medical conditions were the focus of 9.74% of the studies, highlighting their vulnerability to COVID-19 and their concerns about vaccination. Other segments of the population that were included in this review were U.S. parent population (7.17%), pregnant or postpartum women (4.23%), age-specific groups (3.86%), occupational populations (2.94%), and unvaccinated U.S. adults (2.57%).

Most of the studies fell within the 101–500 participant range (28.49%). Smaller studies with 0–100 participants made up 13.79% of the articles. Very large studies with a sample size of over 100,000 participants were also included in this study (2.57%). Some studies (1.47%) did not have a specified sample size (N/A).

Many of the studies included in the analysis (n = 276) were carried out on a nationwide level (Figure 3). From the studies included, 268 of the studies were conducted within individual states in the United States. The studies primarily took place in states with larger populations, indicating significant research-interest densely populated regions. States like these included California (43), New York (25), Texas (17), Florida (15), and Pennsylvania (15). Preferences for denser states may stem from greater accessibility and variability in population demographics. Notably, some investigations concentrated on specific states like Minnesota and Massachusetts, suggesting state-specific concerns or unique challenges related to vaccine hesitancy in those areas. There was a study that took place in Puerto Rico, a territory of the United States, showing the variability in the locations included in this study.

Table 2 describes the vaccine-related characteristics gained from the different studies. The 5C model was another variable used in understanding vaccine hesitancy in this review. The model broke down hesitancy into five variables, each applied to the articles included in this review. Confidence was the most prominent, with 523 occurrences, highlighting its crucial role in trust in the efficacy and safety of the vaccine, as well as the healthcare system’s competence in delivering vaccinations. Collective responsibility (351 occurrences) and calculation (350 occurrences) followed, underscoring the social obligation individuals feel towards community health and the complex consideration of vaccine benefits and risks. Vaccine uptake rates were categorized into percentage ranges. Most of the studies reported 61–70% vaccine uptake (19.85%), followed by 71–80% (17.10%) and 51–60% (14.89%). Some (10.48%) of the studies did not report a vaccine acceptance rate and were labeled as N/A. The lowest uptake rates, 1–10% and 11–20%, each represented less than 1% of the total studies (0.37% and 0.92%). Review of the studies identified common characteristics of vaccine hesitancy. Health concerns were the most common, with 311 occurrences. The speed of the vaccine development/safety concerns followed with 289 occurrences, then mistrust (206), misinformation and misperception (96), and systematic and institutional factors (91). Factors influencing vaccine uptake that are critical to understand the causes of nationwide vaccine hesitancy included trust and confidence (186 instances), community and social factors (133), and demographics and identity (130).

Specific characteristic and vaccine data from each of the 544 studies, including the year of publication, study setting, study design, population, sample size range, reported vaccine uptake rate (%), hesitancy predictors, and uptake factors, can be accessed in the supplementary materials.

## 4. Discussion

Our review identifies health concerns (20.97%), vaccine characteristics (19.49%), and mistrust (13.89%) as the most common factors driving COVID-19 vaccine hesitancy, while trust and confidence (13.18%), community and social factors (9.43%), and demographics and identity (9.21%) promote vaccine uptake. These findings align with the 2021 systematic review by Yasmin et al. [15], which also highlighted demographic factors such as sex, race, age, education, and income as significant determinants of vaccine acceptance. Both reviews underscore the higher hesitancy rates among Black/African Americans and the importance of trust in health authorities (Table A4). Understanding these determinants is crucial for developing targeted interventions to enhance vaccine coverage and public health outcomes.

### 4.1. Hesitancy Predictors

The two most prevalent indicators of vaccine hesitancy identified in this study were health concerns and vaccine characteristics. Health concerns encompass worries about potential side effects, long-term health implications, and overall safety of the vaccine [16]. Addressing these health concerns through transparent communication about vaccine safety profiles, ongoing monitoring, and management of adverse events can help alleviate fears and build confidence in the vaccination process. Our review identified vaccine characteristics as a hesitancy predictor in 19.49% of studies. Previous studies have shown that the four main vaccine characteristics associated with COVID-19 vaccine hesitancy are efficacy, safety, the country of the vaccine manufacturer, and the place of vaccine administration [16]. Additionally, the rapid development and approval process of the vaccines and the types of technology used (e.g., mRNA vaccines) might also play a role in vaccine hesitancy [17]. Public health campaigns should focus on educating the public about the rigorous testing and regulatory processes that vaccines undergo, and the scientific advancements that have facilitated their development without compromising safety or efficacy.

Mistrust in institutions, including the government, pharmaceutical companies, and the healthcare system, was a notable predictor of hesitancy in this review. This mistrust may stem from historical instances of medical malpractice, perceived profit motives, and inconsistent messaging during a pandemic [18,19,20]. Even after two years into the pandemic, mistrust remained high, underscoring the persistent challenges in addressing vaccine hesitancy. Assuming that vaccine hesitancy stems solely from public ignorance and misunderstanding of science is misleading and leads to ineffective educational strategies [21]. This perspective also prevents scientific and governmental institutions from critically examining their own practices regarding earning and maintaining public trust [22]. Efforts to build trust must include engaging with community leaders, ensuring transparency in vaccine development and distribution processes, and addressing specific concerns and misinformation through trusted channels.

The common hesitancy reasons across various vaccines include concerns about side effects, misinformation, and doubts regarding efficacy [23]. For instance, a review on seasonal and pandemic influenza vaccines identified negative attitudes, decreased perceived effectiveness, and lack of trust in health authorities as significant barriers to vaccine uptake [24]. These concerns are echoed in studies on COVID-19 vaccines, where general mistrust in vaccine benefits and safety, concerns about unforeseen effects, and poor compliance with government guidelines were prominent barriers [25].

However, certain factors are uniquely significant to COVID-19 vaccine hesitancy. The politicization of the pandemic, the expedited production and authorization of vaccines, and the massive spread of global misinformation and conspiracy theories have intensified vaccine hesitancy [26]. This is evident from studies reporting significant mistrust and negative attitudes towards COVID-19 vaccines, particularly among ethnic minorities and those from lower socioeconomic backgrounds [25]. Moreover, the variability in COVID-19 vaccine acceptance rates across different regions, with notably lower rates in the Middle East, Eastern Europe, and Russia, further highlights the unique challenges posed by the pandemic [27]. Addressing these concerns through transparent communication, building trust in health authorities, and targeted public health campaigns is crucial for improving vaccine uptake and controlling the pandemic.

A comparison of vaccine hesitancy and uptake between our findings in the United States and Europe reveals both key similarities and differences. Our review shows that in the U.S., vaccine uptake rates were generally between 61 and 70%, with health concerns, safety concerns due to the rapid development of vaccines, and mistrust being the primary predictors of hesitancy. Misinformation and systematic factors also contributed significantly. Similarly, a European study found common determinants of vaccine hesitancy across countries, such as fear of side effects, distrust in vaccine quality, and concerns about the rapid development of vaccines [28]. Both regions showed significant mistrust and misinformation impacting vaccine uptake, and higher education levels were associated with lower hesitancy in both contexts. However, gender differences were more pronounced in Europe, where women were more hesitant than men in five of the eight countries studied [28], whereas various demographic factors influenced vaccine hesitancy in the U.S. Additionally, another European survey found that 11% of respondents were hesitant, perceiving vaccines as risky and poorly tested, while 59% were confident in their effectiveness and safety [29]. These regional nuances highlight the need for tailored public health strategies to address vaccine hesitancy effectively.

### 4.2. Uptake Predictors

Trust and confidence in the vaccine and the entities endorsing it emerged as the most common factor promoting vaccine uptake. Public confidence in vaccines is fundamentally about public trust/trust in the vaccine itself, the healthcare providers administering it, and the policymakers responsible for vaccine provision. Building and maintaining public trust is essential, and this can be achieved through consistent, clear, and evidence-based communication from reliable sources. Moreover, healthcare providers play a crucial role in instilling vaccine confidence, with substantial evidence indicating that physicians, nurses, and other healthcare providers are among the most trusted sources for health information [30].

Community and social factors played a critical role in vaccine uptake among the studies included in this review. Social norms, peer influences, and support from community groups can significantly impact an individual’s decision to be vaccinated. Given the diverse cultural and social landscape of the United States, responses to vaccination campaigns vary widely [7,31]. Public health strategies must therefore be tailored to address these diverse perspectives and foster trust through community engagement and culturally sensitive communication.

Our review identified demographics and identity as an important vaccine uptake factor, where we found that age, gender, ethnicity, and socioeconomic status can influence acceptance of vaccine. Previous research has indicated that vaccine beliefs tend to cluster according to race, education, and socioeconomic background [21]. Studies have shown that parents in higher income brackets often have lower levels of concern about the safety and necessity of vaccines compared with those in lower income brackets [32]. Factors such as parenthood, Black race, lack of prior vaccination, no health insurance, and low perceived disease risk have been associated with decreased rates of vaccine acceptance hurdles [33]. Tailoring communication and outreach efforts to address the unique concerns and barriers faced by different demographic groups can enhance vaccine accessibility and acceptance.

### 4.3. Strengths and Limitations

Our systematic review offers several strengths, particularly its wide geographic coverage to understand vaccine hesitancy. By including studies from various states, both densely and sparsely populated, this review provides a national overview of vaccine attitudes. Additionally, this review provides detailed insights into numerous predictors of vaccine hesitancy and factors promoting uptake, such as health concerns, mistrust, and community influences. The inclusion of diverse study designs and populations, particularly web-based studies, ensures the capture of broad perspectives during the pandemic and highlights the most common influences driving both hesitancy and uptake. However, there are notable limitations in this review. The concentration of studies in larger more populous states may limit the generalizability of findings to rural or less populated areas. One of the limitations of our study is that, despite the recent Advisory Committee on Immunization Practices (ACIP) recommendation on 28 February 2024 that all persons aged ≥65 years receive one additional dose of any updated COVID-19 vaccine (i.e., Moderna, Novavax, or Pfizer-BioNTech) [34], our analysis did not adequately represent vaccine hesitancy in this age group. This insufficient representation prevented a focused sub-analysis on this particularly vulnerable population. Future research should specifically target these groups to better understand their unique concerns and barriers to vaccination. The application of the 5C model across diverse contexts was challenging, as many studies did not explicitly address these constructs, leading to subjective mapping. Additionally, some studies did not report vaccine uptake rates, creating gaps in the data and potential bias in understanding vaccination trends. The 5C model’s focus on psychological factors may overlook significant cultural, socioeconomic, and structural influences on vaccination behavior. These theoretical and practical challenges underscore the need for a more integrative approach when assessing vaccination behavior across different settings.

Lastly, the reliance on web-based surveys might exclude populations with limited internet access, thereby not fully representing all demographic groups

## 5. Conclusions

Our review underscores the multifaceted nature of vaccine hesitancy and acceptance. By understanding and mitigating the predictors of hesitancy and reinforcing the factors that encourage uptake, we can improve vaccination rates and advance public health objectives. Future research should continue to explore these dynamics and develop tailored strategies that resonate with diverse populations, ultimately fostering a more robust and resilient public health response to COVID-19 and beyond.

## Figures and Tables

**Table 1:** Study characteristics of included studies.

Variables	Category	Frequency	Percentage
Year of publication	2022	320	58.82%
	2023	224	41.18%
Study setting	Web-based	384	70.59%
	In-person survey	77	14.15%
	Hybrid	45	8.27%
	Telephone-based survey	38	6.99%
Study design	Cross-sectional survey	399	73.35%
	Mixed method design	44	8.09%
	Qualitative studies	37	6.80%
	Longitudinal studies	42	7.72%
	Miscellaneous *	22	4.04%
Population	U.S. adults	176	32.35%
	Ethnic and racial minority populations	80	14.71%
	Healthcare workers	65	11.95%
	People with specific medical conditions	53	9.74%
	U.S. parents	39	7.17%
	University population	25	4.60%
	Pregnant or postpartum women	23	4.23%
	Age-specific studies	21	3.86%
	Occupational population	16	2.94%
	Unvaccinated U.S. adults	14	2.57%
	Military personnel	11	2.02%
	Social services populations	6	1.10%
	Gender-specific populations	5	0.92%
	Inmate population	3	0.55%
	People positive for COVID-19	3	0.55%
	Low- and middle-income U.S. adults	3	0.55%
	Religious or faith-based populations	1	0.18%
Sample size range	0–100	75	13.79%
	101–500	155	28.49%
	501–1000	82	15.07%
	1001–5000	144	26.47%
	5001–10,000	21	3.86%
	10,001–50,000	35	6.43%
	50,001–100,000	10	1.84%
	>100,000	14	2.57%
	N/A	8	1.47%

* Observational, ecological, descriptive studies.

**Table 2:** Vaccination-related characteristics of reviewed studies.

Variables	Category	Frequency	Percentage
5C Model	Confidence	523	32.91%
	Complacency	221	13.91%
	Convenience	145	9.13%
	Calculation	350	22.03%
	Collective responsibility	350	22.03%
Reported Vaccine Uptake Rate (%)	1–10%	2	0.37%
	11–20%	5	0.92%
	21–30%	25	4.60%
	31–40%	35	6.43%
	41–50%	36	6.62%
	51–60%	81	14.89%
	61–70%	108	19.85%
	71–80%	93	17.10%
	81–90%	72	13.24%
	91–100%	30	5.51%
	N/A	57	10.48%
Hesitancy Predictors	Health Concerns	311	20.97%
	Speed of Vaccine Development/Safety Concerns	289	19.49%
	Mistrust	206	13.89%
	Misinformation/Misperception	96	6.47%
	Systemic and Institutional Factors	91	6.14%
	Political and Ideological	76	5.12%
	Psychological Factors	69	4.65%
	Racial and Ethnic Influences	61	4.11%
	Social Influence	49	3.30%
	Demographics and Identity	44	2.97%
	Psychosocial Factors	41	2.76%
	Cultural Beliefs	31	2.09%
	Accessibility Issues	27	1.82%
	Economic Factors	26	1.75%
	Individual Experiences	19	1.28%
	Communication and Messaging	15	1.01%
	Technological Aspects	10	0.67%
	Environmental Factors	9	0.61%
	Global and Local Dynamics	5	0.34%
	Legal and Ethical Considerations	3	0.20%
	N/A	3	0.20%
	Compliance and Convenience	2	0.13%
Uptake Factors	Trust and Confidence	186	13.18%
	Community and Social Factors	133	9.43%
	Demographics and Identity	130	9.21%
	Healthcare Provider Recommendations	117	8.29%
	Health Concerns	113	8.01%
	Psychological Factors	92	6.52%
	Information Sources and Education	92	6.52%
	External Motivations and Support	81	5.74%
	Vaccination History	68	4.82%
	Previous Experiences and Behavior	65	4.61%
	Safety	60	4.25%
	Political and Social Influences	52	3.69%
	Vaccine Choice	45	3.19%
	Policy and Communication	42	2.98%
	Media and Information Influence	26	1.84%
	N/A	23	1.63%
	Ending Pandemic	24	1.70%
	Job Security	18	1.28%
	Mandate	16	1.13%
	Incentives and Rewards	14	0.99%
	Availability	12	0.85%

## Data Availability

Not applicable.

## Supplementary Materials

The following supporting information can be downloaded at: https://www.mdpi.com/article/10.3390/vaccines12070747/s1.

## Appendix A

**Table A1:** Data sources.

Host Database	Database/Collection	Article Count
Proquest	Public Health Database	5086
	Biological Science Collection	4000
	Health and Medical Collection	2831
	Psychology Database	1046
Ebscohost	Medline	2883
	CINAHL	1758
	Health Source: Nursing/Academic Edition	577
	APA PsycINFO	236
	Psychology and Behavioral Sciences Collection	91
	APA Psycarticles	21
NLM	PubMed	2706
Web of Science	Web of Science	2595
Elsevier	Science Direct	1337
	Scopus	410
Google Scholar	Google Scholar	1006
Wiley Online Library	Wiley Online Library	780
Springerlink	Springerlink	610
Wiley	Cochrane COVID-19 Study Register	497
ITHAKA	JSTOR	68
Wiley	Cochrane Library	22

**Table A2:** MeSH terms.

Host Database	Database/Collection	MeSH Terms	Additional Search Options
Proquest	Public Health Database	(COVID-19 OR COVID-19 OR covid OR coronavirus OR 2019-ncov OR SARS-CoV-2 OR COVID-19) AND (vaccin* hesitancy OR vaccin* refusal OR the anti-vaccine movement OR immunization refusal OR unvaccinated) AND (us OR united states OR united states of america OR usa OR u.s. OR america OR american OR u.s.a. OR u.s.)	Publication Date: January 2022–August 2023 PEER (yes)
	Biological Science Collection		
	Health and Medical Collection		
	Psychology Database		
Ebscohost	Medline		Publication Date: January 2022–August 2023
	CINAHL		
	Health Source: Nursing/Academic Edition		
	APA PsycInfo		
	Psychology and Behavioral Sciences Collection		
	APA Psycarticles		
Pubmed	Pubmed		
Clarivate	Web of Science	((TS = (COVID-19 or COVID-19 or covid or coronavirus or 2019-ncov or SARS-CoV-2 or COVID-19)) AND TS = (vaccine hesitancy or vaccine refusal or the anti-vaccine movement or immunization refusal or unvaccinated)) AND TS = (us or united states or united states of america or usa or u.s. or america or american or u.s.a. Or u.s.)	Publication Years: 2022–2023
Elsevier	Science Direct	COVID-19, vaccination hesitancy, united states	Years: 2022–2023 Document Type: Article
	Scopus		
Google Scholar	Google Scholar	COVID-19, vaccination hesitancy, united states	Sorted by date, Removed articles published before 2022
Wiley	Wiley Online Library	COVID-19, vaccination hesitancy, vaccine acceptance, united states	Years: 2022–2023
Springerlink	Springerlink	COVID-19, vaccination hesitancy, united states	Filters Applied: Article, 2022–2023
Wiley	Cochrane COVID-19 Study Register	Vaccine and hesitancy and united and states and covid and 19	Created: 1 January 2022–31 August 2023
ITHAKA	JSTOR	COVID-19, vaccination hesitancy, united states	Date: 2022–2023
Wiley	Cochrane Library		

vaccin* incorporates vaccine, vaccines, vaccinate and vaccination.

**Table A3:** Materials summary.

Item Type	Count
Journal Articles	27,932
Report	197
Book Section	152
Conference Paper	66
Newspaper Article	57
Preprint	51
Blog Post	35
Thesis	27
Magazine Article	19
Book	18
Case	6

**Table A4:** Population studies with vaccination acceptance rates.

Population Studied	Acceptance Rate (Avg)
Arab	74.35%
Asian	66.7%
White	62.9%
Minority Population	60.07%
Hispanic	57.59%
Black/African-American	56.07%
